# Language specificity in cortical tracking of speech rhythm at the mora, syllable, and foot levels

**DOI:** 10.1038/s41598-022-17401-x

**Published:** 2022-08-05

**Authors:** Varghese Peter, Sandrien van Ommen, Marina Kalashnikova, Reiko Mazuka, Thierry Nazzi, Denis Burnham

**Affiliations:** 1 MARCS Institute for Brain Behaviour and Development, Western Sydney University, Penrith, NSW Australia; 2 School of Health and Behavioural Sciences, University of the Sunshine Coast, Sippy Downs, Australia; 3 Integrative Neuroscience and Cognition Center, CNRS-Université Paris Cité, Paris, France; 4 Neurosciences Fondamentales, University of Geneva, Geneva, Switzerland; 5 BCBL, Basque Center on Cognition, Brain and Language, San Sebastian, Guipuzcoa Spain; 6 IKERBASQUE, Basque Foundation for Science, Bilbao, Bizcaya Spain; 7 Laboratory for Language Development, RIKEN Center for Brain Science, Saitama, Japan; 8 Department of Psychology and Neuroscience, Duke University, Durham, NC USA

**Keywords:** Cognitive neuroscience, Language

## Abstract

Recent research shows that adults’ neural oscillations track the rhythm of the speech signal. However, the extent to which this tracking is driven by the acoustics of the signal, or by language-specific processing remains unknown. Here adult native listeners of three rhythmically different languages (English, French, Japanese) were compared on their cortical tracking of speech envelopes synthesized in their three native languages, which allowed for coding at each of the three language’s dominant rhythmic unit, respectively the foot (2.5 Hz), syllable (5 Hz), or mora (10 Hz) level. The three language groups were also tested with a sequence in a non-native language, Polish, and a non-speech vocoded equivalent, to investigate possible differential speech/nonspeech processing. The results first showed that cortical tracking was most prominent at 5 Hz (syllable rate) for all three groups, but the French listeners showed enhanced tracking at 5 Hz compared to the English and the Japanese groups. Second, across groups, there were no differences in responses for speech versus non-speech at 5 Hz (syllable rate), but there was better tracking for speech than for non-speech at 10 Hz (*not* the syllable rate). Together these results provide evidence for both language-general and language-specific influences on cortical tracking.

## Introduction

**Figure 1:**
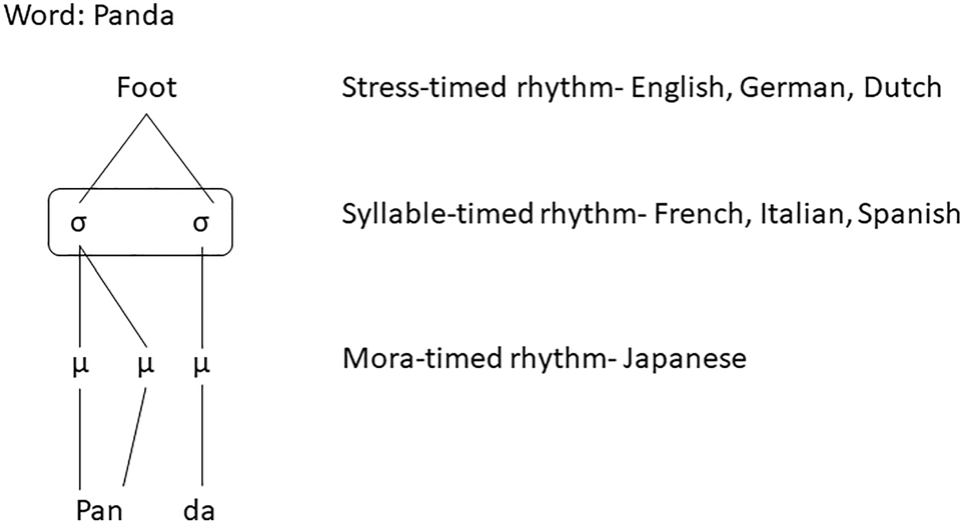
The word ‘Panda’ in terms of its feet (1), syllables (2) and morae (3).

Spoken language is rhythmic, and while one aspect of this rhythm is language-general due to the universal consonant/vowel alternations at the syllable level and cyclical opening and closing of the jaws^1^, speech rhythm also differs across languages^2^. At the linguistic level, three distinct rhythm classes of languages have been proposed: stress-based, e.g., English, syllable-based, e.g., French, and mora-based, e.g., Japanese^3–5^. These classes differ in their basic rhythmic units: the supra-syllabic foot in stress-timed languages, the syllable in syllable-timed languages, and the sub-syllabic mora in mora-timed languages. The relationship between these three units is illustrated for the word ‘panda’ in Fig. 1. These three language-specific rhythmic units occur at different frequencies in the speech signal of each language. The supra-syllabic foot occurs at a rate of about 2–2.5 Hz^6,7^, the syllable at about 5 Hz^8^, and the sub-syllabic mora at about 8–10 Hz^9,10^. Yet, because of the language-general jaw cycles to produce syllables, the syllabic rhythm is considered more fundamental, such that syllabic information should dominate the amplitude envelope of speech. In this regard, Ding et al.^8^ found a peak in the envelope spectrum of 4.3–5.4 Hz across nine languages with typologically different rhythmic structures, although some cross-linguistic differences in various measures of rhythmicity have been reported in the literature^11,12^.

Given the complex nature of speech rhythm, the present study investigates whether and how language-general and language-specific properties of speech result in processing differences in listeners of the three rhythmic classes. In syllable-timed languages, language-specific syllabic processing by the listener may combine with the language-general syllable-level (+ acoustic at 5 Hz, + linguistic at 5 Hz), resulting in augmented processing at 5 Hz. In stress- or mora-timed languages, the language-specific nature of processing speech may be at odds with the language-general syllable-level information, resulting in decreased processing at 5 Hz for both stress-timed (+ acoustic at 5 Hz, − linguistic at 5 Hz, + linguistic at 2.5 Hz) and mora-timed (+ acoustic at 5 Hz, − linguistic at 5 Hz, + linguistic at 10 Hz) languages.

With respect to language-specific rhythms, rhythmic classes appear to have psychological reality right from birth, albeit in rather coarsely-defined classes, and behavioral data suggest that infants and adults are sensitive to both language-general and language-specific rhythm. Indeed, newborn infants discriminate between languages across stress-, syllable-, and mora-timed classes but not between languages from within those classes^13^, and recent evidence suggests newborns have perceptual biases for prosodic grouping based on their specific in utero language experience^14,15^. Language rhythm perception goes on to aid the segmentation of word forms from the acoustically-continuous speech stream during the first year of life: use of stress information in US English^16^ and Dutch^17^; use of syllable information in French^18,19^. Language-specific rhythm continues to aid speech segmentation across the lifespan, assisting adults to segment the speech stream and access words, and adults have established language-specific procedures in the use of rhythm cues: native French language listeners use syllable but not stress information to access words, whereas native English language listeners use stress but not syllable information^20,21^; and native Japanese adults use mora but not syllable information to access words, whereas native French listeners use syllable but not mora information^22,23^.

With respect to language-general rhythms, there is also evidence for the possibility of a language-general, acoustic, priority for processing speech at the syllable level, at least in infancy. First, Japanese-learning infants’ use of moraic-based information to segment the speech stream is likely weaker than English- and French-learning infants’ use of stress and syllable information, since Japanese-learning infants do not discriminate monomoraic from bimoraic syllables until 10 months of age, suggesting that the mora may be a less prominent rhythmic unit than the foot or the syllable^24,25^. Additionally, French-born newborns discriminate words that differ in number of syllables, but not in number of morae^26^, suggesting either a general syllable advantage, or further language-specific rhythmic processing right from birth. Second, while stress-based segmentation seems to emerge around 7.5 to 10 months (in US English^16^; Dutch^17,27^), syllable-based segmentation is found as early as 4 months in French-learning infants^28^, suggesting the syllable might be a privileged/default rhythmic unit. Less is known on language-general rhythm processing in adults (despite the dominance of syllable rate information in the amplitude envelope^8^), and how it might interact with language-specific processing.

To explore this issue, we focus on cortical tracking of the speech signal. The term ‘cortical tracking’ refers to the adjustment of an internal quasi-periodic system (neural oscillations) to match the phase of an external periodic or quasi-periodic stimulus (speech rhythm). By tracking the rhythmicity of speech, the high-excitability phase of neural oscillations comes to match informative features in the speech envelope, increasing speech intelligibility, while phases of low excitability are aligned with irrelevant information^29–32^. Studies that assume or argue that cortical tracking involves synchronisation of ongoing neural oscillations often use the term ‘neural entrainment’ rather than cortical tracking^33^. As our study emphasizes psycholinguistic issues, and we are agnostic about the underlying neural mechanism, we will generally use the term cortical tracking throughout.

There are various accounts of cortical tracking, focusing either on top-down or bottom-up mechanisms. For example, one top-down proposal posits that the right hemisphere prefers oscillations in the delta-theta rates (1–10 Hz, prosodic and syllabic variations) whereas the left hemisphere prefers oscillations at the gamma rate (25–45 Hz, phoneme-level variations^34^). Lack of such hemispheric asymmetries is thought to relate to atypical language development^7,35–37^. Moreover, cortical tracking of speech also appears strongly modulated by top-down cognitive functions such as attention^30,31,38,39^. In contrast, evidence for bottom-up effects comes from studies showing cortical tracking of the envelope of both speech and non-speech sounds^40–44^. Other studies suggest that these bottom-up effects do not result from envelope tracking, but rather the tracking of discrete acoustic landmarks such as amplitude peaks at the centres of vowels, or acoustic edges caused by peaks in the rate of amplitude envelope change^45,46^. Hence, evidence suggests both bottom-up and top-down influences on cortical tracking, which might extend to rhythm processing. Moreover, it is important to note that there may be concurrent entrainment to different levels of information in speech processing: Ding et al.^47^ showed that in response to connected speech in which the envelope contained only amplitude modulations at word boundaries, cortical activity is found at different timescales concurrently, tracking different linguistic structures such as words, phrases and sentences. So, while syllable rate information may dominate the speech amplitude envelope, other information might well be of importance in the cortical tracking of speech and subsequent behavioural processes.

### This study

We know that different languages have different dominant rhythmic patterns; that there is a seemingly language-general concentration of acoustic energy at the 5 Hz syllable level; and that there is cortical tracking of continuous speech. However, no previous study has directly investigated how experience with a particular language rhythm might modulate cortical tracking, and how these experiential differences might interact with the language-general dominance at 5 Hz (syllable-level). If we are to understand how speech rhythms are encoded, and whether, and if so, how, the syllable might play a crucial role in this encoding, it is necessary to compare neural encoding of speech by speakers of languages that differ in their rhythm. In this comparative psycholinguistic study, the aim is to investigate whether the dominant rhythmic unit of the listeners’ own language affects speech processing, more specifically, whether cortical tracking of a neutral speech envelope is modulated by the different rhythmic patterns of different languages, the foot, syllable or mora. To this end, we tested adult native listeners of stress-based Australian English (an English variety that shares rhythmic properties with US and British English^48,49^), syllable-based French, and mora-based Japanese on their cortical tracking of a common speech envelope which allowed for coding at the foot, syllable, and mora levels. We created synthetic speech stimuli that were phonotactically legal in all three languages and that could be perceived to have stress-, syllable- or mora-based rhythm and recorded cortical tracking responses to amplitude envelopes at three peak frequencies: 2.5 Hz (foot level), 5 Hz (syllable level) and 10 Hz (mora level). Two competing hypotheses and a third hybrid hypothesis were considered as follows.

#### Stimulus-based (language-general) tracking hypothesis

Cortical tracking is determined only by low-level information. No cross-linguistic differences are expected and syllable rate information should dominate.

#### Language-specific tracking hypothesis

Cortical tracking is modulated by language-specific structures. Adults with backgrounds in languages with different rhythmic classes should track at different language-specific rates, i.e., English listeners should track best at 2.5 Hz, French listeners at 5 Hz, and Japanese listeners at 10 Hz.

#### Hybrid hypothesis (most likely given above evidence)

There is basic, language-general cortical tracking at 5 Hz, but response strength at the different rhythmic levels (2.5 Hz for stress, 5 Hz for syllable, and 10 Hz for mora) is influenced by the particular rhythmic structure of the listener’s native language.

These hypotheses were tested via two sets of comparisons: cross-language comparisons, and speech/non-speech comparisons.

##### Cross-language comparisons

We compared English, French, and Japanese adult listeners on their cortical tracking, at 2.5, 5, and 10 Hz, of neutral speech envelopes—in English, French and Japanese synthesised voices—that all allowed processing at the foot (2.5 Hz), syllable (5 Hz), and mora (10 Hz) frequencies. The stimulus-based processing hypothesis predicts no cross-linguistic differences. The language-specific hypothesis predicts 2.5 Hz tracking should be best in English listeners, 5 Hz tracking in French listeners, and 10 Hz tracking in Japanese listeners. The hybrid hypothesis predicts similar cross-linguistic differences, plus a bias for better cortical tracking at 5 Hz in all three language groups: hence the 5 Hz advantage for the French listeners might be stronger than the 2.5 Hz advantage for the English listeners, and the 10 Hz advantage for the Japanese listeners.

##### Speech/non-speech comparisons

We compared the three language groups on their cortical tracking, at 2.5, 5, and 10 Hz, of a neutral speech envelope in a voice in an unfamiliar-to-all language, Polish, and a vocoded non-speech equivalent of the Polish voice. The stimulus-based tracking hypothesis predicts better tracking at 5 Hz than at 2.5 Hz or 10 Hz, and no speech/non-speech processing difference at any frequency. The language-specific tracking hypothesis predicts a speech over non-speech superiority, which would possibly be enhanced at the language-specific frequency for each language group compared with the other two frequencies. The hybrid hypothesis predicts a speech over non-speech superiority, which may be enhanced at the language-general 5 Hz rate.

## Method

### Ethics

The ethics committee for Human Research and Western Sydney University (Approval number: H9660) and the Ethics Committee of CERES, France (No. 2011-14) approved the experimental methods used in the study. All experiments were performed in accordance with relevant guidelines and regulations. Informed consent was obtained from all the participants.

### Participants

There were three groups of participants, native speakers of (i) Australian English (n = 24), (ii) French (n = 26) and (iii) Japanese (n = 24). The sample size is comparable with the studies that used similar methodology to investigate cross linguistic effects on cortical tracking of language^47,50^. The experiment was conducted in Sydney, Australia for the English- and Japanese-speaking participants and in Paris, France for the French-speaking participants. English-speaking participants were recruited from the Western Sydney University community, French-speaking participants via an online participant recruitment system, and Japanese-speaking participants from advertisements placed in a website for Japanese visitors to Australia on temporary work visas. None of the participants were fluent in the other two languages, although some had learned one of the languages in school, but it remained a foreign language for all. Even though the Japanese participants were tested in Sydney, their level of English-language proficiency was poor, and they had minimal exposure to the English stress rhythm as they all had lived in Australia for less than 1 month (mean = 18.56 days; range 7–30 days). Note that if anything, knowledge of the other languages should reduce our predicted cross-linguistic differences. All participants reported normal hearing and no history of psychological or language disorders. Data from 1 English, 1 French and 4 Japanese participants were removed as they were contaminated by artifacts. The final sample was 23 English-, 25 French- and 20 Japanese-speaking participants.

### Stimuli

As three different language groups were tested, it was essential to choose speech stimuli that would not favour processing in any one language group. One possibility would be to use natural speech from three native speakers, one for each language, or from a balanced trilingual speaker. Neither was advisable—the three speakers would need to be equated on a large number of articulatory variables, and finding a balanced trilingual speaker was unlikely. Therefore, synthetic speech was required. In speech synthesis the central portion of a single segment is the most stable while the transition from one segment to another contains the richest information and thus is the most difficult to model. Diphone synthesisers, in which each half-phoneme is concatenated with each other half-phoneme, overcome this problem to a large extent by cutting the synthesis units at points of relative stability—within rather than between the phonemes. MBROLA is a diphone synthesiser in which such concatenation has been completed for a large number of voices, with independent imposition of appropriate prosody including intonation, duration, and shift in spectral quality. In this way, different voices can be given the same prosodic overlay^51,52^. We used four such MBROLA voices, one English, one French, and one Japanese and, with respect to examining speech/non-speech differences, a fourth, Polish voice (and its non-speech vocoded equivalent, see below) sampled at 16,000 Hz. The voices themselves were not important per se, what was important and what was maintained across the four voices and the vocoded voice was the common speech envelope allowing coding at the foot, syllable, or mora level.

All three language groups were presented with the same three voices, English, French, and Japanese, to ensure that all participants' responses were measured on the same stimuli, one of which was synthesized in their native language and the other two in non-native languages. Yet, in the analysis of cross-language comparisons, we combined the responses to the three voices, because they were all created by the same MBROLA diphone synthesis procedure, plus the three voices being analysed together provided greater generalisability of any findings regarding the processing of that common speech envelope. In the analyses of Polish and its vocoded equivalent, these similar two ‘voices’ were compared to examine speech/non-speech differences.

The stimuli were CVn (consonant–vowel-nasal) monosyllables. The vowels, consonants and nasals used in the CVn combinations were present in English, French, Japanese, and Polish. Ten consonants (/p/, /t/, /k/, /b/, /d/, /g/, /s/, /z/, /ʃ/,/j/) three vowels (/i/, /a/, /ɔ/) and three nasals (/m/, /n/, /ŋ/) were used to create 14 phonotactically possible target syllables. When the first consonant was a stop, that consonant was 20 ms in duration and the following vowel was 80 ms in duration. For fricative and affricate onsets, both consonants and vowels were 50 ms in duration. The nasal was always 100 ms in duration. The syllables were synthesised in four different Mbrola voices (us1-English, fr4-French, jp2-Japanese and pl1-Polish; https://github.com/mbrola/mbrola-voices) with the fundamental frequency (F0) of 180 Hz. The noise-vocoded non-speech versions of the Polish voice was created by extracting the broadband envelope of the syllables between 0–120 Hz and modulating white noise in the envelope. This 1-channel noise vocoded stimuli can be considered non-speech as previous studies have shown that speech identification scores for such stimuli is close to 0%^53^.

The syllables were concatenated into 30-s-long sequences, each containing 150 syllables. The sequences were created dynamically in Presentation 18.1 (https://www.neurobs.com) during the experiment with the constraint that the same syllable did not occur in succession. Ten syllables (out of 14) were repeated 11 times (randomly selected) and the remaining 4 were repeated 10 times to create sequences of 30-s duration. Eight unique sequences were created for each of the five conditions (4 Mbrola voices and vocoded speech, see Online Supplementary Material for examples of spectrograms) for every participant during the experiment. Two additional sequences were created in which either one or two syllables in the sequence had an F0 of 280 Hz. Participants were instructed to press a button when they heard a pitch change. EEG responses for these two target sound sequences were not analysed. Participants from all the groups listened to the same set of stimuli.

### Sound sequence analysis

While our stimuli were constructed so that the foot, syllable and mora levels could all be perceived, it should be noted that our sequences were simplified compared to complex natural speech, so that there were no acoustic/prosodic cues to the foot level (contra what is found in natural speech), all our syllables had the same structure (CVn), and we used only two types of morae (CV and n). Therefore, to determine the rhythmic structure present at the acoustic level, the envelopes of the different 30-s-long sequences were extracted using the Hilbert function implemented in MATLAB (MathWorks, Natick, MA, USA) to obtain a time-varying estimate of the instantaneous amplitude of the sound envelope. The obtained waveforms were then transformed in the frequency domain using a discrete Fourier transform to obtain a frequency spectrum of envelope magnitude. This analysis yielded a model of response corresponding to the envelope of acoustic energy of the sequences. As the stimuli sequences were created by concatenating 200 ms duration syllables, the envelope amplitude at 5 Hz was more prominent than at 10 Hz. However, there was no peak evident at 2.5 Hz, which could affect foot-level perception, although illusory perception of the foot in the absence of acoustic cues has been found in adult speakers of another stress-based language, German^54^. Hence, in these stimuli, the syllable level appears as the more pronounced. In contrast, our predicted cross-linguistic differences would only emerge at the foot level if the English-speaking adults show illusory foot level perception, and at the mora-level if the Japanese-speaking adults are able to extract moraic structure from our strict alternations of CVs and Ns.

### EEG recording, EEG analysis, and determination of EEG peaks

The recording conditions were identical across the two testing locations (Sydney and Paris), using the same equipment, same stimuli (English, French, Japanese, Polish and vocoded Polish) and same calibration of the intensity of the stimuli. The EEG was recorded using the 129-channel (128 channels plus reference electrode at Cz) Hydrocel Geodesic Sensor Net (HCGSN), the NetAmps 300 amplifier and NetStation 4.5.7 software (EGI Inc.) at a sampling rate of 1000 Hz with the reference electrode placed at Cz. The electrode impedances were kept below 50 kΩ. The continuous EEG was saved for offline analyses.

The recorded EEG was analysed offline using fieldtrip^55^ (version: 20200409), EEGLAB^56^ (version: 2019.1) and letswave 6 (https://www.letswave.org/) toolboxes in MATLAB 2017a. Multiple toolboxes were used for the analysis since different toolboxes have different features and strengths in EEG analysis (filtering and bad channel rejection were performed in fieldtrip; ICA, re-referencing and time domain averaging were performed in EEGLAB; all the subsequent analyses were performed in letswave). The EEG was not downsampled for any of the analyses.

The EEG was first band pass filtered between 0.3–30 Hz (using a windowed sync finite impulse response filter) and then divided into epochs between 0 to 30 s relative to sound sequence onset. Noisy EEG channels were identified by visual inspection and removed (average: 3 channels; range 0–12). Independent component analysis (ICA) was then performed on the EEG data and components with stereotypical features of eye blinks and eye movements removed (average 3.1 components: range 2–6). The removed EEG channels were then interpolated using spherical spline interpolation. Epochs with amplitude exceeding ± 100 μV were removed. Participants with less than six good epochs out of 8 for each condition (75% good trials) were rejected (1 English, 1 French and 4 Japanese participants). Epochs were then re-referenced to the average of all the electrodes. Epochs were averaged separately for each condition. The time domain averaging procedure was used to enhance the signal to noise ratio in the EEG responses time-locked to the input sequence.

**Figure 2:**
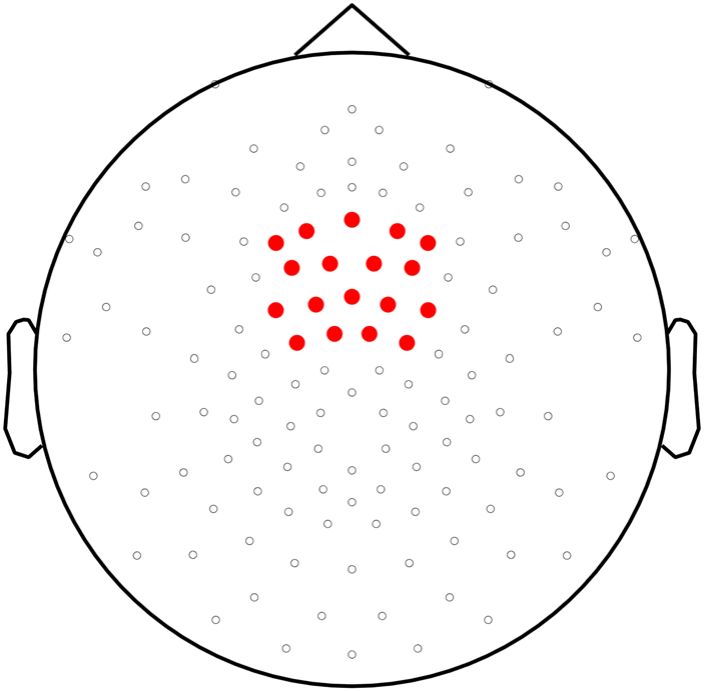
Location of 128 electrodes in the HCGSN. The highlighted electrodes were used for analysis.

The averaged waveforms were then transferred to the frequency domain using discrete Fourier transform, producing a spectrum of EEG amplitudes between 0–500 Hz with a frequency resolution of 0.025 Hz. To further increase the signal to noise ratio, at each bin of the frequency spectra, the average amplitude measured at neighbouring frequency bins (2nd to 12th frequency bins relative to each bin) was subtracted for each participant, condition, and electrode. This process relies on the assumption that, in the absence of a periodic EEG response, the signal amplitude at a given frequency bin should be similar to the signal amplitude of the mean of the surrounding frequency bins^57–60^. In line with previous studies on auditory perception^60,61^, the magnitude of the response at the foot (2.5 Hz), syllable (5 Hz) and mora (10 Hz) rates was computed by averaging the responses at 10 fronto-central electrodes (Fig. 2). These electrodes were selected as previous studies have found stronger entrainment for auditory stimuli at fronto-central electrodes^60^.

To determine whether significant EEG responses were elicited at the frequencies of interest, z-scores were calculated at the target frequencies (2.5, 5 and 10 Hz) for each stimulus type for each language group. The z-score at a given frequency was computed as the difference in amplitude between that frequency and the mean of the 20 neighbouring frequency bins (10 on either side), divided by the standard deviation of the 20 neighbouring bins. The 20 neighbouring bins represented a frequency range of 0.50 Hz (0.25 Hz on either side) and excluded the two immediately adjacent frequency bins^62,63^. As in these previous studies, we considered z scores greater than 3.1 (p < 0.001, one-tailed, i.e., signal > noise) to be significant. These scores are reported in Table S1 in the Online Supplementary Materials (Table S1).

**Figure 3:**
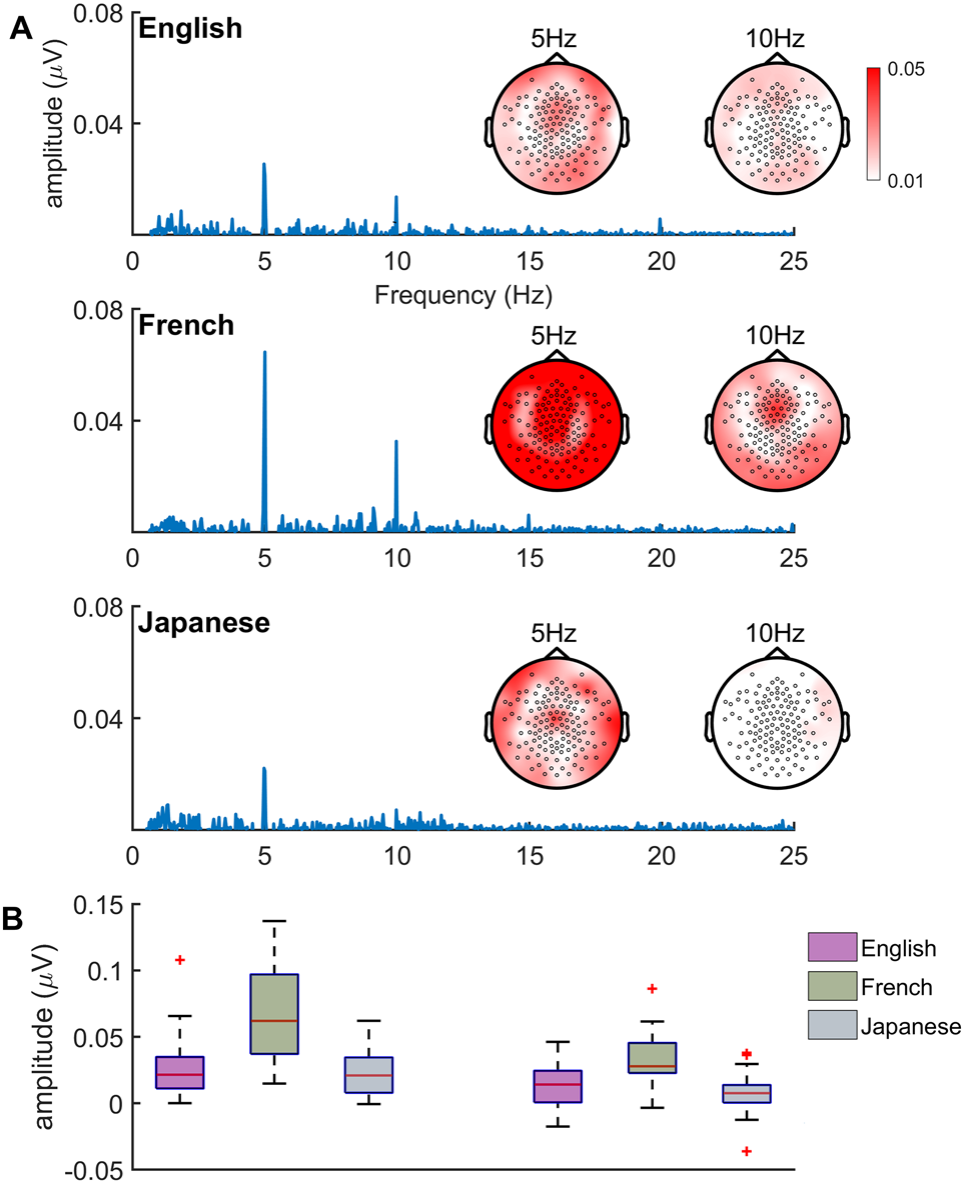
(**A**) Spectrogram and topography of the responses for the Speech stimuli (English, French and Japanese) across the three groups—English, French and Japanese language adults. (**B**) Response amplitude at 5 Hz and 10 Hz.

**Figure 4:**
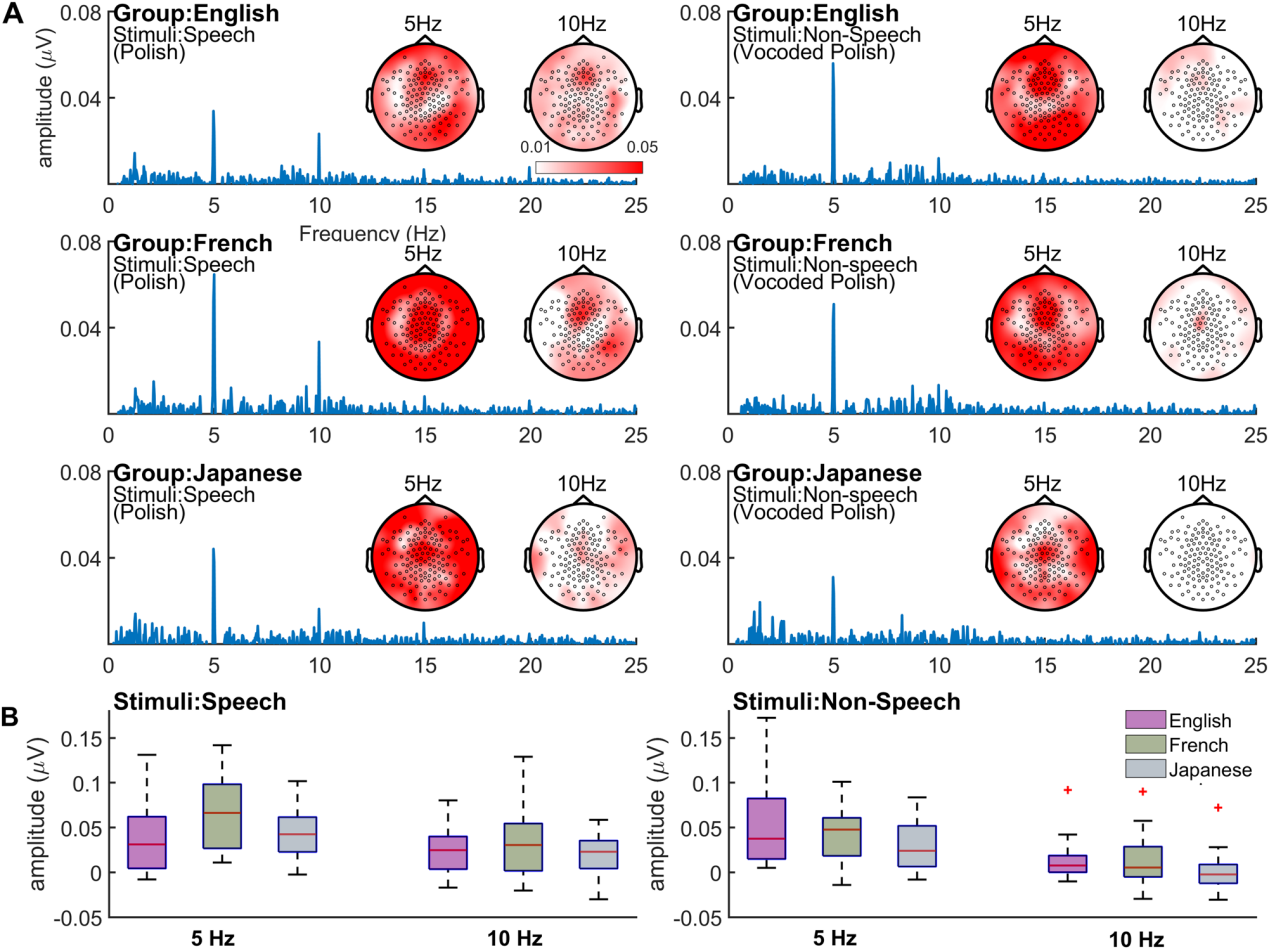
(**A**) Spectrogram and topography of responses for Speech (Polish) and non-speech (Vocoded Polish) stimuli across the three groups—English, French and Japanese language adults. (**B**) Response amplitude at 5 Hz and 10 Hz.

At 2.5 Hz, no significant responses were found. At 5 Hz, all three groups showed significant responses for all stimuli. At 10 Hz, English and French groups showed significant response to all stimuli whereas the Japanese group showed significant response only to Polish stimuli (Central tendency and variation values are shown for 5 and 10 Hz in Figs. 3 and 4). As the z-scores showed there were significant EEG peaks at 5 Hz and 10 Hz, but not at 2.5 Hz, statistical analyses were conducted for the 5 Hz and 10 Hz data but not for the 2.5 Hz data.

### Statistical analysis

Linear Mixed Effects (LME) models were fitted to the data. The continuous variable (Amplitude) was scaled and centred around zero to assist with model convergence, and simple coding was used for all the categorical variables (Group, Frequency, and Condition). Analyses were conducted in R using the lme4 package for conducting the LME analyses^64^ and the lmerTest package to calculate p-values (using the Satterthwaite approximation) and to conduct post hoc pairwise comparisons^65^. All models were first constructed including the maximal random effects structure^66^, and random slopes were removed until achieving model convergence. As a result, all models presented here only include random intercepts for participants.

## Results

The grand averaged EEG spectra are shown in Figs. 3 and 4. The two sets of LME analyses were conducted to compare cortical tracking by the three language groups (English, French, Japanese) at the two frequencies for which there were significant EEG peaks (5 and 10 Hz), one set for each of the two aims of the experiment: (1) *Cross-language Comparisons,* in Model 1 and (2) *Speech versus Non-Speech* in Models 2a, 2b, and 2c. Model results are summarised in the text and the detailed model outputs are presented in the Online Supplementary Materials.

### Model 1: cross-language comparisons

Model 1 assessed the effects of Frequency (5 Hz, 10 Hz) and Group (French, English, Japanese) and their interaction on response amplitudes and is specified as follows:

$$ {\text{Amplitude}}\sim {\text{Frequency}} + {\text{Group}} + {\text{Frequency }} \times {\text{ Group}} + ({1}|{\text{Participant}}). $$

Mean and individual data that are the basis of Model 1 analyses are plotted in Fig. 3B, and detailed model output is presented in the Online Supplementary Materials (Table S2). There was a main effect of Frequency, F(1, 2902.7) = 60.989, p < 0.001, and a main effect of Group, F(2, 65.84) = 7.585, p = 0.001. There was also a significant Frequency by Group interaction, F(2, 2902.7) = 5.908, p = 0.003, and follow up pairwise comparisons were conducted at each frequency. At 5 Hz, the French produced higher responses than both the English (β = − 0.355, SE = 0.087, t = − 4.100, CI [− 0.526, − 0.183], p < 0.001) and the Japanese (β = 0.375, SE = 0.091, t = 4.103, CI [0.194, 0.556], p < 0.001), while the English–Japanese difference was not significant. At 10 Hz, the French produced higher responses than the Japanese, β = 0.214, SE = 0.091, t = 2.347, CI [0.033, 0.396], p = 0.021, but the French–English and English–Japanese differences failed to reach significance. Additional within-group comparisons showed that the main effect of Frequency, with higher 5 Hz than 10 Hz responses, was present in the French, β = 0.424, SE = 0.056, t = 7.538, p < 0.00, English, β = 0.142, SE = 0.060, t = 2.351, p = 0.019, and Japanese groups, β = 0.264, SE = 0.067, t = 3.941, p < 0.001.

### Model 2a, speech versus non-speech

Mean and individual data that are the basis of Model 2 analyses are plotted in Fig. 4B, and detailed model outputs (2a, 2b, 2c) are presented in Online Supplementary Materials (Tables S3, S4, S5). Model 2a assessed the effect of Frequency (5 Hz, 10 Hz), Group (French, English, Japanese), and Stimulus [Speech (Polish) vs. Non-Speech (Vocoded)], and their three-way interaction on response amplitudes, and is specified as follows:

$$ {\text{Amplitude}}\sim {\text{Frequency}} + {\text{Group}} + {\text{Stimulus}} + {\text{Frequency }} \times {\text{ Group }} \times {\text{ Stimulus}} + ({1}|{\text{Participant}}). $$

There was a main effect of Frequency, F(1, 1933.55) = 59.616, p < 0.001, and main effects of Group, F(2, 65.65) = 7.646, p = 0.001, and Stimulus, F(1, 1941.11) = 9.553, p = 0.002. There was also a significant three-way interaction, F(2, 1933.55) = 3.125, p = 0.044, which was explored further by separate models conducted at 5 and 10 Hz, and in the case of Group by Stimuli interactions, by pairwise comparisons of responses to Speech and Non-Speech stimuli within each language group.

### Model 2(b), speech–non-speech, 5 Hz

The 5 Hz model yielded no main effects of Group or Stimulus, but there was a significant Group by Stimulus interaction, F(2, 937.54) = 3.639, p = 0.027, which was explored by pairwise comparisons for Stimulus. All three pairwise comparisons failed to reveal significant speech/non-speech differences.

### Model 2 (c), speech–non-speech, 10 Hz

The 10 Hz model yielded main effects of Group, F(2, 998) = 7.589, p = 0.001, and Stimulus, F(1, 998) = 10.748, p = 0.001, but no Group by Stimulus interaction. The French produced larger responses than both the English, β = − 0.207, SE = 0.074, t = − 2.781, CI [− 0.353, − 0.061], p = 0.006, and the Japanese, β = 0.289, SE = 0.079, t = 3.673, CI [0.135, 0.445], p < 0.001, with no significant English/Japanese difference, and across groups, responses were higher for speech than for non-speech stimuli, β = − 0.209, SE = 0.064, t = − 3.278, CI [− 0.335, − 0.084], p = 0.001.

### ERPs

To determine whether the group differences found above could be attributable to testing locations (Sydney versus Paris), amplitude of the N1 ERP response to the onset syllable was analysed from the same electrodes. We did not find any significant differences in the N1 amplitude for testing location, suggesting that the physical differences between the testing locations did not affect the responses. For a detailed description of the results, please see Online Supplementary Materials.

## Discussion

The aim of this study was to investigate the effect of language-general and language-specific influences on adults’ cortical tracking of speech envelopes. To that end, three hypotheses were tested: whether cortical tracking is based on (i) low-level, stimulus-based, language-general rhythmic information, (ii) the dominant rhythmic unit of the listener’s native language, or (iii) some combination, a hybrid, of these. To test these hypotheses, adults from three language backgrounds were tested: English with the foot as the dominant rhythmic unit (at a frequency of 2.5 Hz), French with the syllable (5 Hz), and Japanese with the mora (10 Hz). Thirty-second-long sequences of CVn syllables were presented in speech envelopes in four different synthesised voices, English, French, Japanese and Polish, and a non-speech (vocoded) equivalent of the Polish voice, sequences that could all be tracked at 2.5 Hz (corresponding to the foot), 5 Hz (the syllable) or 10 Hz (the mora). Cortical tracking at 2.5, 5, and 10 Hz was investigated.

To test for significant EEG peaks at the three frequencies of interest, z-scores at those and surrounding frequencies revealed significant EEG peaks at 5 and 10, but not 2.5 Hz. Hence, the hypotheses could only be tested at 5 and 10 Hz. Two complementary sets of analyses were conducted: (a) cross-language comparisons of English, French and Japanese adults’ cortical tracking of the English, French, Japanese voices at 5 and 10 Hz, (b) a speech/non-speech comparison of tracking the Polish voice and its non-speech equivalent at 5 and 10 Hz.

### Cross-language comparisons

The stimulus-based tracking hypothesis would predict better tracking at 5 Hz by all three language groups, and the language-specific tracking hypothesis would predict better tracking at 5 Hz by the French than the other two groups, and better tracking at 10 Hz by Japanese than the other two groups. Linear mixed effects analysis of cortical tracking by the three language groups at 5 Hz and at 10 Hz revealed that response amplitudes were significantly greater at 5 Hz than at 10 Hz for all three language groups. This supports the stimulus-based tracking hypothesis that tracking should be best at the 5 Hz language-general syllable rate. However, inspection at each frequency showed that at 5 Hz, response amplitudes by the French listeners were significantly greater than those of either the English or Japanese; whereas at 10 Hz, the French still showed the highest response amplitudes, but this superiority was not as strong (see Fig. 3B). As 5 Hz is the frequency corresponding to the syllable, the dominant rhythmic unit in French, the group difference at 5 Hz supports the language-specific hypothesis at least for the French language background adults. If the language-specific hypothesis were to be more generally supported, then it would be expected that at 10 Hz the Japanese language adults should show superior tracking. This expectation was not supported. However, the present results at 10 Hz—a decrease in the superiority of the French adults’ tracking (which should be a non-preferred rate for French listeners) over that of the English and Japanese—support, to some extent, the language-specific hypothesis pattern of results, again only for the French adults.

So, there is support for the stimulus-based tracking hypothesis (5 Hz > 10 Hz responses for all three groups), and some support for the language-specific hypothesis, at least for the French group (French responses greater than English and Japanese at 5 Hz, and not so dominant at their non-preferred rate of 10 Hz). Together, these results provide partial support for the hybrid hypothesis of joint language-general stimulus-based, and language-specific determinants of cortical tracking.

### Speech versus non-speech

The stimulus-driven, language-general hypothesis would predict better tracking at 5 Hz than at 10 Hz (a) irrespective of language background, i.e., no interaction with language group (specifically no superiority for the French over the other two groups, and (b) irrespective of stimulus, i.e., no interaction with speech/non-speech (specifically no superiority for speech over non-speech), since there was higher amplitude at 5 than 10 Hz in all stimuli. The language-specific hypothesis would predict better tracking for speech than for non-speech and especially enhanced speech > non-speech effects for the French at 5 Hz and for the Japanese at 10 Hz.

Linear mixed effects model analysis of cortical tracking by the three language groups at 5 and 10 Hz for speech and non-speech revealed that overall, there were greater response amplitudes at 5 Hz than at 10 Hz, in line with the stimulus-based tracking hypothesis; and overall, there were greater response amplitudes for speech than for non-speech, in line with the language-specific tracking hypothesis (see Fig. 4B). However, a three-way interaction of 5 Hz/10 Hz × Speech/Non-Speech × Language Group revealed that at 5 Hz there were no significant Speech/Non-Speech differences and no between language group differences. On the other hand, at 10 Hz there was a Speech > Non-Speech effect, which was equally strong across all three language groups, and overall better tracking by the French than by the English or Japanese adults over both stimulus conditions.

There was better tracking at 5 than 10 Hz and the superior tracking at 5 Hz was undifferentiated (no effect of speech versus non-speech, or language group). This supports the stimulus-based tracking hypothesis; cortical tracking is best when determined by low-level syllable rate information (which is present in all our stimuli, as verified in our acoustic analyses), and is so irrespective of whether this information is speech or not, and irrespective of the language background (and dominant rhythmic unit) of the listener. However, at 10 Hz, there were consistent speech > non-speech effects and language background effects similar to those in the cross-language comparisons (French adults showing better tracking than English and Japanese adults), results that support the language-specific tracking hypothesis. Taken together, these findings support our hybrid hypothesis of joint stimulus-based, language-general and language-specific determinants of cortical tracking.

### Tests of hypotheses

Together neither the results from the cross-language nor from the speech/non-speech analyses provide unequivocal evidence for either the stimulus-based, (language-general) or the language-specific tracking hypotheses, since there is evidence for both the involvement of stimulus-based, language-general cortical tracking in the overall better tracking at 5 than 10 Hz, as well as for language-specific modulation of tracking due to the dominant rhythmic unit of the listener’s native language. As argued above, these results thus provide some evidence for the hybrid hypothesis. However, the evidence for modulation of tracking due to language background rests mainly on the superior tracking results for the French listeners at 5 Hz and their reduced superiority at 10 Hz. Future studies are required to continue this work to investigate whether there is evidence for superior tracking of the foot in English and of the mora in Japanese. One way to proceed could be by using stimuli that better instantiate these two levels compared to the syllabic level. For the foot level, this would mean using stimuli in which there is an alternation of prosodically strong and weak syllables; for the mora level, this would mean using a larger range of mora types besides the postvocalic nasals used here, such as lengthened vowels and geminated consonants.

Before concluding, we would like to address two further points of our findings, namely (1) the link between the acoustic properties of our stimuli and responses obtained, and (2) the link between the responses at 5 and 10 Hz.

Regarding the link between the acoustic properties of our stimuli and cortical tracking, it should be noted that the larger responses at 5 Hz compared to 10 Hz, and the lack of response at 2.5 Hz follow the strength in peaks revealed by our acoustic analysis (highest peak at 5 Hz, smaller peak at 10 Hz, and no peak at 2.5 Hz). This parallel in strength can be seen as support for the stimulus-based tracking hypothesis. However, the stimulus-based hypothesis would not predict cross-linguistic differences in tracking the same signal, as found here. Indeed, the French showed superior tracking at 5 Hz (the frequency which coincides with their dominant rhythm unit of their native language) than the English and Japanese. Moreover, the response at 10 Hz was also modulated according to the native language. Tracking was significantly higher for the French listeners, followed by the English and the Japanese. Both cross-linguistic modulations indicate language-specific determination of cortical tracking besides stimulus-based determination, again favouring a hybrid hypothesis of the origin of cortical tracking.

Regarding the link between the responses observed at 5 and 10 Hz, it should be noted that because the sound envelope we used as stimuli are not pure sinewaves, we know from prior studies that the elicited periodic EEG responses to these stimuli are also not pure sinewaves, so that in the frequency domain representation of this response, a series of peaks at the expected frequencies and upper harmonics are expected^67^. Hence, the response at 10 Hz could be a harmonic of the 5 Hz response. Although the crosslinguistic modulation of the 5 and 10 Hz responses differ, we decided to test this possibility post hoc using a Linear Mixed Effects model that regressed the effect of Group and each participant’s 5 Hz response on the 10 Hz response amplitude to the speech stimuli (see Online Supplementary Materials Table SI6 for model specification and output). This model yielded a main effect of Group, *F*(2, 67.3) = 3.624, *p* = 0.03, reflecting our main analyses, but no effect of 5 Hz response amplitude (*p* = 0.175). This suggests that the possibility of the 10 Hz response being a harmonic of the 5 Hz response cannot entirely account for our findings. Moreover, since the 10 Hz response is not solely a harmonic reflection of 5 Hz, its crosslinguistic modulation (effect of Group) provides further evidence (in addition to the evidence for the French at 5 Hz) for linguistically-influenced tracking, and thus for our hybrid hypothesis.

## Conclusion

This study has shown that the greatest and most consistent level of enhanced cortical tracking for all three language groups is at the 5 Hz level, which corresponds to the language-general acoustically-determined speech rhythm. In addition, in the speech/non-speech comparison, response amplitudes are consistently higher for all three language groups at 5 Hz than 10 Hz. These results strongly suggest the involvement of low-level acoustic determinants in cortical tracking. Nevertheless, the fact that this cortical tracking at the 5 Hz level is greatest for the French, for whom the 5 Hz syllable rate is the dominant rhythmic unit, suggests augmentation of low-level acoustic determinants by higher-level linguistic determinants. The possible involvement of such higher-level linguistic determinants is corroborated by the speech/non-speech results in which there is no speech/non-speech effect at the basic 5 Hz frequency, but speech > non-speech effects for each language group at the non-basic level, 10 Hz, frequency.

Together these results show that there are low-level acoustic determinants of cortical tracking of speech, but these are not independent of higher-level linguistic determinants. This supports a hybrid view of the acoustic and linguistic determinants of cortical tracking. Further research is required to investigate (i) the nature of linguistic determinants across languages, especially in stress-timed and mora-timed languages, and (ii) the developmental trajectory of any language-specific processing in infants and children from languages differing in their dominant rhythmic unit, again those languages examined here: English (foot, 2.5 Hz), French (syllable, 5 Hz) and Japanese (mora, 10 Hz).

## Supplementary Information

Supplementary Information.

## Data Availability

The datasets generated during the current study are available from the corresponding author on reasonable request.
