# Supplementary material for: Language specificity in cortical tracking of speech rhythm at the mora, syllable, and foot levels
Source: Sci Rep. 2022 Aug 5;12:13477. doi: 10.1038/s41598-022-17401-x (PMC9356059; doi:10.1038/s41598-022-17401-x)
Supplement: Supplementary file 1 — Supplementary Information. [file 41598_2022_17401_MOESM1_ESM.pdf]

Online Supplementary Materials

Language-specific speech processing:
Cortical tracking to speech rhythm at mora, syllable, and foot levels

Table SI1. Z scores computed to determine whether significant EEG responses were elicited at the frequencies of interest (2.5, 5 and 10 Hz) for each stimulus type for each language group. Asterisks (*) represent significant z scores (z scores > 3.1; p < .001, one-tailed, i.e., signal > noise).

Group	Voice Condition	2.5 Hz	5 Hz	10 Hz
English	Speech (French, English, Japanese)	-0.64	9.83*	4.75*
	Speech (Polish)	-0.25	13.31*	8.56*
	Non-speech (Vocoded Polish)	-1.49	18.94*	4.26*
French	Speech (French, English, Japanese)	0.27	21.21*	7.25*
	Speech (Polish)	-0.01	10.08*	8.24*
	Non-speech (Vocoded Polish)	-0.34	11.84*	3.26*
Japanese	Speech (French, English, Japanese)	0.69	8.20*	2.17
	Speech (Polish)	-1.02	12.62*	3.84*
	Non-speech (Vocoded Polish)	1.12	8.96*	0.25

Table SI2. Detailed output of Linear Mixed Effects Model 1: Cross-Language Comparisons that assessed the effects of Frequency (5Hz, 10 Hz) and Group (French, English, Japanese) and their interaction on response amplitudes [Amplitude ~ Frequency + Group + Frequency * Group + (1|Participant)]

	ß	*SE*	*t*	*p*
(Intercept)	-.009	.006	0.142	.887
Frequency [10]	-.142	.006	-2.351	.019
Group [French]	.355	.009	4.1	<.001
Group [Japanese]	-.020	.009	-0.215	.831
Frequency [10] × Group [French]	-.282	.008	-3.417	.001
Frequency [10] × Group [Japanese]	-.122	.009	-1.349	.177

Table SI3. Detailed output of Linear Mixed Effects Model 2a: Speech versus Non-Speech

Detailed output of Linear Mixed Effects Model 2a, Speech versus Non-Speech that assessed the effect of Frequency (5Hz, 10Hz), Group (French, English, Japanese), and Stimulus (Speech (Polish) vs. Non-Speech (Vocoded)), and their three-way interaction on response amplitudes [Amplitude ~ Frequency + Group + Stimulus + Frequency * Group* Stimulus + (1|Participant)]

	ß	*SE*	*t*	*p*
(Intercept)	.210	.080	2.599	.010
Frequency [10]	-.579	.105	-5.502	<.001
Group [French]	-.103	.111	-0.009	.993
Group [Japanese]	-.260	.120	-2.16	.031
Stimuli [Speech]	-.169	.105	-1.608	.108
Frequency [10] × Group [French]	.290	.144	2.007	.045
Frequency [10] × Group [Japanese]	.212	.157	1.346	.178
Frequency [10] × Stimuli [Speech]	.452	.148	3.047	.002
Group [French] × Stimuli [Speech]	.313	.144	2.178	.029
Group [Japanese] × Stimuli [Speech]	.373	.157	2.377	.018
Frequency [10] × Group [French] × Stimuli [Speech]	-.467	.203	-2.298	.022
Frequency [10] × Group [Japanese] × Stimuli [Speech]	-.439	.222	-1.981	.048

Table SI4. Detailed output of Linear Mixed Effects Model 2(b): Speech - Non-Speech, 5 Hz that assessed the effect of Group (French, English, Japanese) and Stimulus (Speech (Polish) vs. Non-Speech (Vocoded)), and their two-way interaction on 5Hz response amplitudes [Amplitude ~ Group + Stimulus + Group* Stimulus + (1|Participant)]

	ß	*SE*	*t*	*p*
(Intercept)	0.120	0.091	2.188	.030
Group [French]	0.012	0.126	0.097	.923
Group [Japanese]	-0.247	0.136	-1.815	.072
Stimuli [Speech]	-0.163	0.099	-1.639	.102
Group [French] × Stimuli [Speech]	0.304	0.136	2.241	.025
Group [Japanese] × Stimuli [Speech]	0.357	0.148	2.405	.016

Table SI5. Detailed output of Linear Mixed Effects Model 2(c), Speech - Non-Speech, 10 Hz that assessed the effect of Group (French, English, Japanese) and Stimulus (Speech (Polish) vs. Non-Speech (Vocoded)), and their two-way interaction on 10Hz response amplitudes [Amplitude ~ Group + Stimulus + Group* Stimulus + (1|Participant)]

	ß	*SE*	*t*	*p*
(Intercept)	-0.365	0.077	-4.732	<.001
Group [French]	0.281	0.106	2.656	.008
Group [Japanese]	-0.051	0.115	-0.446	.656
Stimuli [Speech]	0.279	0.109	2.577	.010
Group [French] × Stimuli [Speech]	-0.148	0.149	-0.998	.319
Group [Japanese] × Stimuli [Speech]	-0.063	0.162	-0.391	.696

Table SI6. Detailed output of the post hoc Linear Mixed Effects Model that assessed the effect of Group (French, English, Japanese) and 5 Hz response amplitude on 10Hz response amplitudes to Speech stimuli [Amplitude 10Hz ~ Group + Amplitude 5Hz + (1|Participant)]

	ß	*SE*	*t*	*p*
(Intercept)	0.016	0.052	0.316	.753
Amplitude [5Hz]	0.036	0.027	1.356	.175
Group [French]	0.058	0.072	0.807	.423
Group [Japanese]	-0.144	0.077	-1.857	.068

N1 amplitude

Table S17: Mean, standard deviation (SD) and standard error (SE) of N1 amplitudes

Location	N	Mean	SD	SE
Sydney	43	-1.23	0.97	0.15
Paris	25	-1.31	1.04	0.21

One-way ANOVA on N1 amplitude with the factor Location (Sydney, Paris) did not show any significant effects $F(1,67)=0.93$, $p=.761$.

Stimuli: English

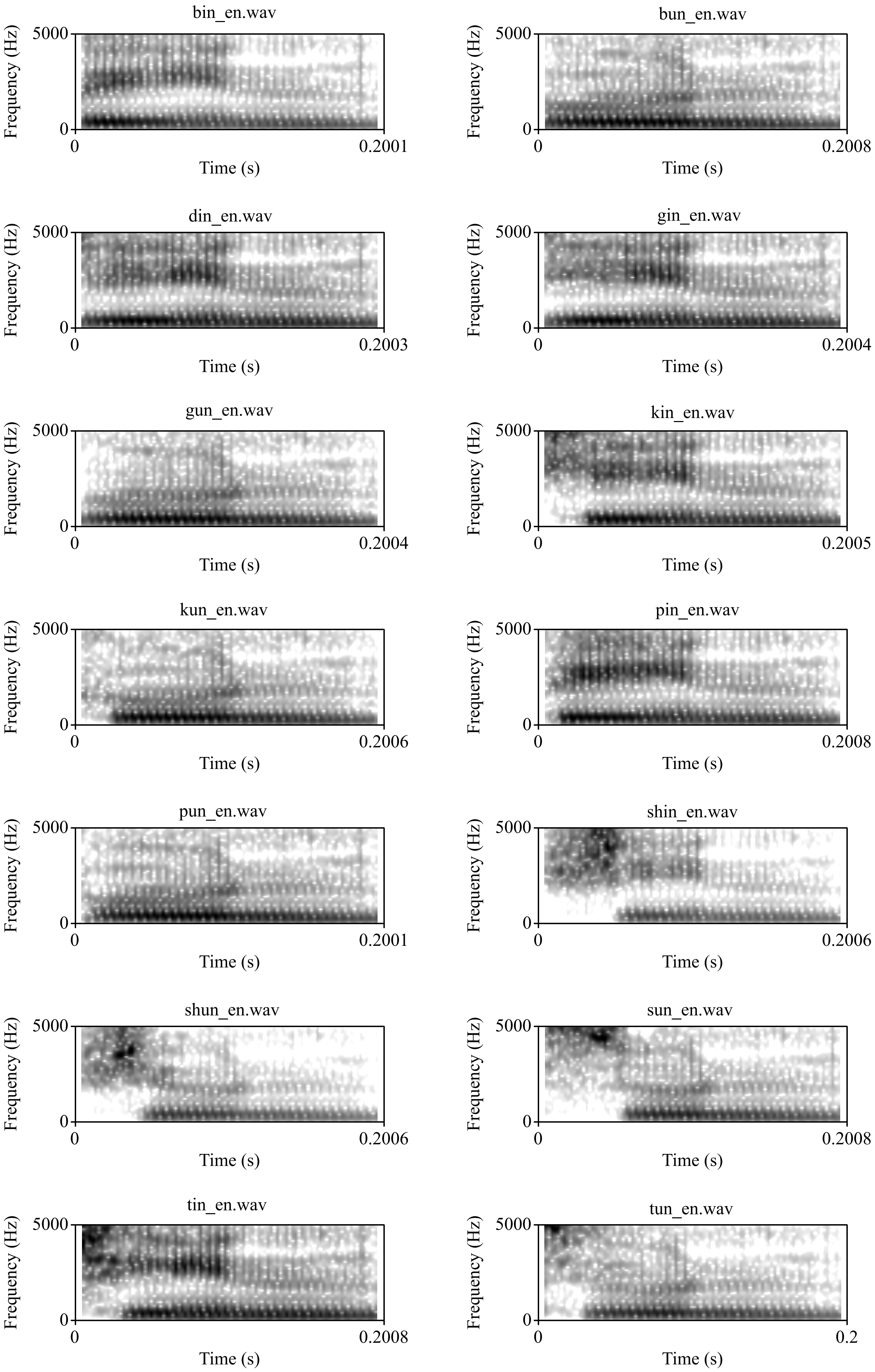

Stimuli: French

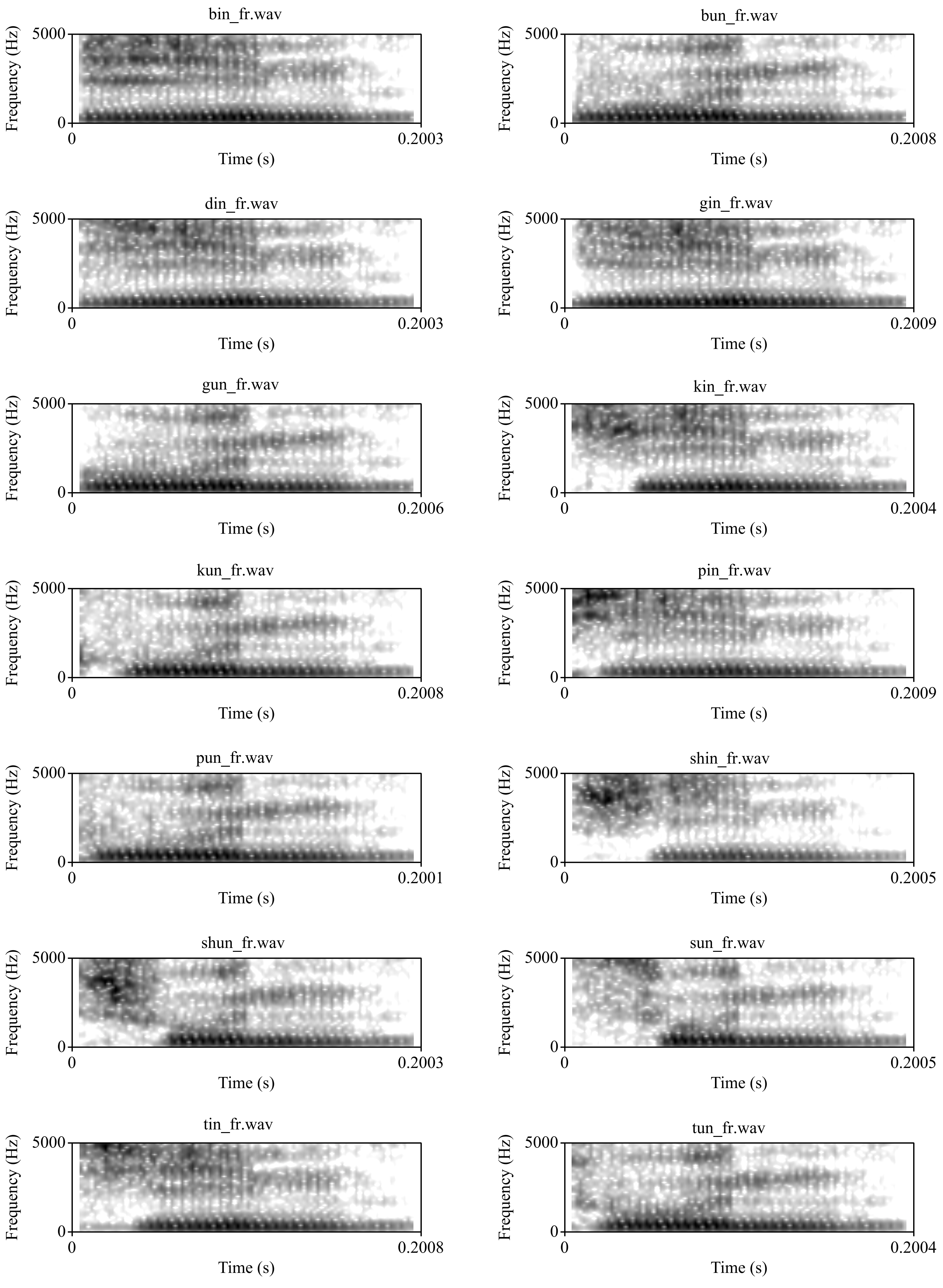

Stimuli: Japanese

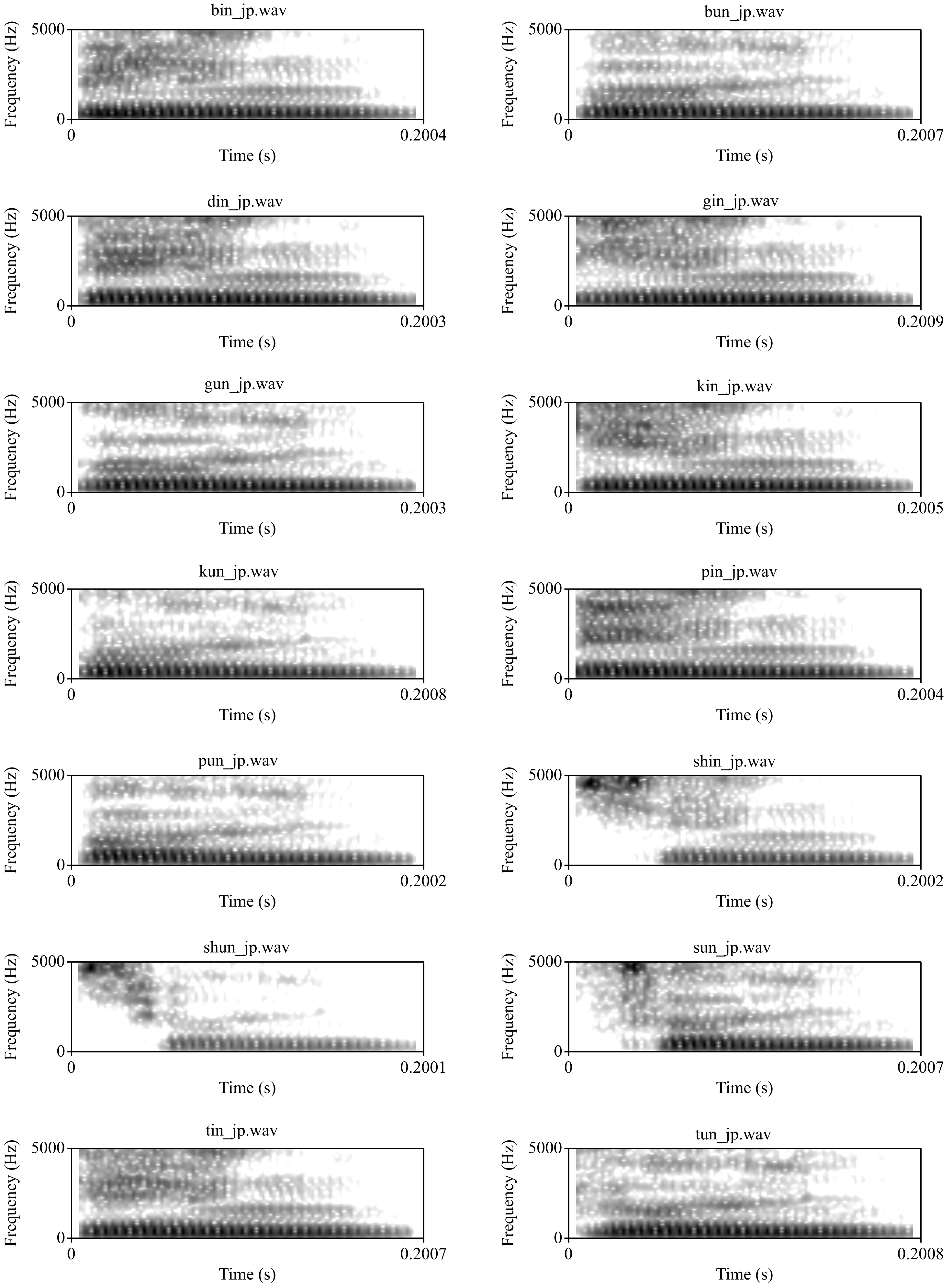

Stimuli: Polish

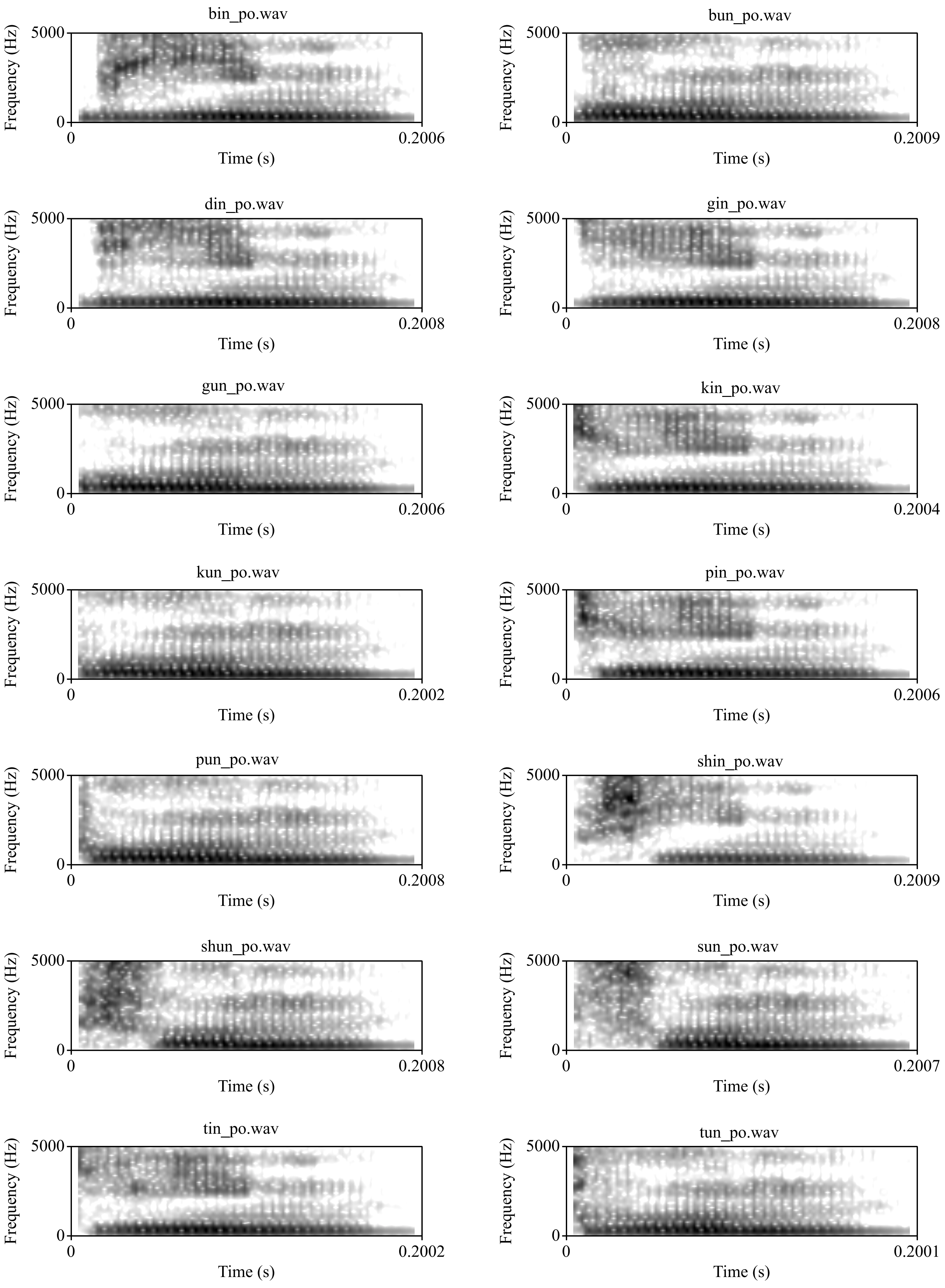

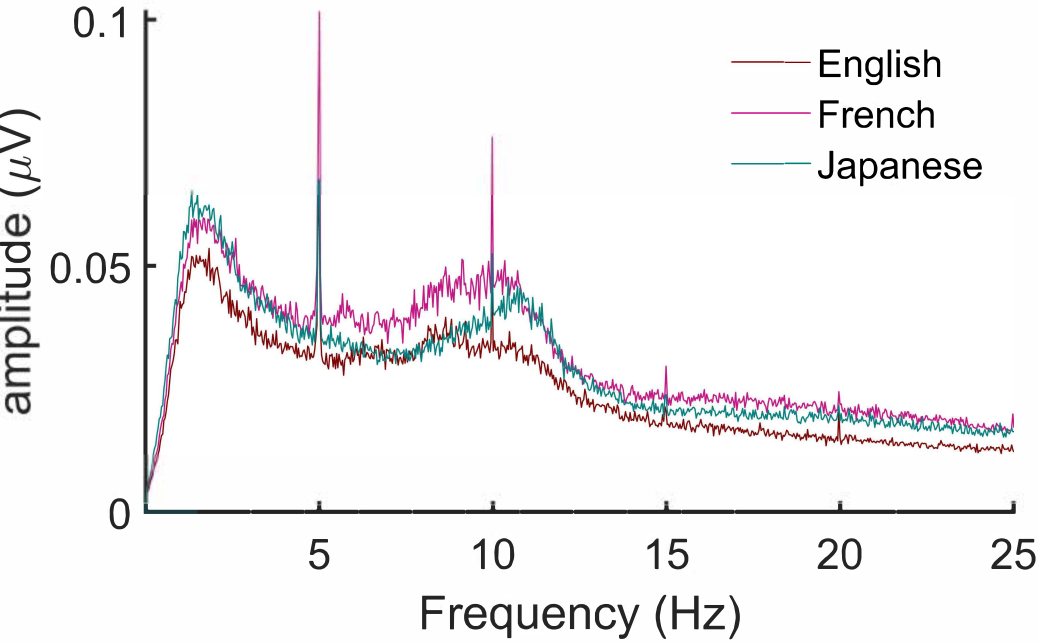

Supplementary Figure: The EEG spectra across the groups (before removal of the baseline)
